# Effects of Different Housing Systems during Suckling and Rearing Period on Skin and Tail Lesions, Tail Losses and Performance of Growing and Finishing Pigs

**DOI:** 10.3390/ani11082184

**Published:** 2021-07-23

**Authors:** Anita Lange, Michael Hahne, Christian Lambertz, Matthias Gauly, Michael Wendt, Heiko Janssen, Imke Traulsen

**Affiliations:** 1 Department of Animal Sciences, Livestock Systems, Georg-August-University, Albrecht-Thaer-Weg 3, 37075 Göttingen, Germany; imke.traulsen@uni-goettingen.de; 2 Clinic for Swine, Small Ruminants, Forensic Medicine and Ambulatory Service, University of Veterinary Medicine Hannover, Foundation, Bischofsholer Damm 15, 30173 Hannover, Germany; Michael.Hahne@tiho-hannover.de (M.H.); Michael.Wendt@tiho-hannover.de (M.W.); 3 Faculty of Science and Technology, Free University of Bozen-Bolzano, Piazza Università 5, 39100 Bolzano, Italy; christian.lambertz@fibl.org (C.L.); matthias.gauly@unibz.it (M.G.); 4 Chamber of Agriculture of Lower Saxony, Mars-la-Tour-Straße 6, 26121 Oldenburg, Germany; heiko.janssen@lwk-niedersachsen.de

**Keywords:** farrowing systems, early socialization, rearing in the farrowing pen, tail biting, average daily gain

## Abstract

**Simple Summary:**

Weaning involves multiple stressors and is one of the most critical periods for piglets. It is known that pigs try to compensate stressful events by different coping strategies that might culminate in the biting of other pigs. The reduction in the number of stressors by optimizing housing conditions might be a way to reduce tail biting, one huge challenge in modern pig production. Since tail docking as a measure to avoid injuries is banned by EU regulations, this study aims to present alternatives to combat tail biting. The present work shows that the group housing of lactating sows and their litters improves pig welfare after regrouping events in terms of skin lesions. Rearing in the farrowing pen with reduced regrouping positively affected the incidence of tail lesions and losses of undocked pigs. Against expectations, free farrowing and group housing systems had no negative impact on later performance during rearing or fattening.

**Abstract:**

Feasible alternatives to stressful weaning and tail-docking are needed to inhibit tail biting. Therefore, we investigated the effects of housing systems for 1106 pigs that were weaned from: (1) conventional farrowing crates (FC), (2) free-farrowing pens (FF), or (3) group housing of lactating sows (GH) into (1) conventional rearing pens (Conv) or (2) piglets remained in their farrowing pens for rearing (Reaf). Tails were docked or left undocked batchwise. All pigs were regrouped for the fattening period. Pigs were scored for skin lesions, tail lesions and losses. After weaning, Conv-GH pigs had significantly less skin lesions than Conv-FC and Conv-FF pigs. After regrouping for fattening, Reaf-GH pigs had significantly less skin lesions than Conv pigs, Reaf-FC and Reaf-FF. The frequency of tail lesions of undocked Conv pigs peaked in week 4 (66.8%). Two weeks later, Reaf undocked pigs reached their maximum (36.2%). At the end of fattening, 99.3% of undocked Conv pigs and 43.1% of undocked Reaf pigs lost parts of their tail. In conclusion, the co-mingling of piglets during suckling reduced the incidence of skin lesions. Rearing in the farrowing pen significantly reduced the incidence of tail lesions and losses for undocked pigs. No housing system negatively affected the performance.

## 1. Introduction

Weaning is one of the most critical periods for piglets, causing an alteration of their welfare, health and performance [1]. While natural and seminatural weaning happens gradually in a time span of up to 17 weeks [2], piglets under conventional conditions are normally weaned at 3 to 4 weeks of age (Council Directive 2008/120/EC). In Europe, the piglets are usually undergoing several steps at weaning: besides separation from the sow, they are relocated from the farrowing environment to rearing pens, with a change in feed and regrouping with unfamiliar piglets (aiming for rearing groups of homogeneous weight) on the same day. Although studies on aggression in (weaning) pigs date back to the 1970s, aggression after mixing is still a common problem in pig husbandry [3,4]. Already, Friend et al. [5] showed that the extent of intermixing of a group of pigs directly affected the number of fights. Fights that arose to establish the hierarchy increased the severity of the skin lesion score, especially in pigs that were regrouped during fattening period [6]. The early socialization of piglets from different litters before weaning resulted in positive effects for later social behavior [4,7,8,9]. For example, when these pigs were grouped, the new hierarchy was established sooner than in groups of less socialized pigs resulting in less skin lesions. These findings were confirmed by a previous study of Lange et al. [10] who investigated the effects of housing systems during suckling and wean-to-finish-rearing on post-weaning stress 24 h after weaning and relocation in terms of skin lesions, serum cortisol and aggressive behavior. Several studies show that loose-housing of sows during lactation, even in groups, can be a feasible alternative to confined housing systems: free farrowing not only improved the welfare of the sow, but also positively influenced the piglets in terms of behavior and performance [11,12,13]. Piglets under natural conditions or in single loose-housing farrowing pens experience a lot of nose-to-nose contacts with their mother sow instantly after birth and before the sow intended to lie down [14,15]. Piglets from single loose-housed sows dealt differently with their post-weaning environment (more playful and exploratory behavior), suggesting differences in maternal behavior between loose-housed sows and sows in farrowing crates [12]. Single loose-housed sows have the chance to interact with their piglets, possibly enhancing the maternal and also social behavior of the piglets [13] even after weaning. Still, the group housing of sows and their litters often raise concerns about cross-suckling and the impaired weight gain of the piglets due to interrupted suckling bouts [16,17]. In general, a better performance at weaning was shown to enhance weight gain at later stages [18]. Since weaning involves multiple stressors [1], the weaning process should be optimized to better prepare the piglets for the rearing and finishing period. It is discussed that pigs try to compensate stressful events by different coping strategies that might result in the oral manipulation of the environment and pen mates, possibly culminating in the biting of other pigs [19,20]. The reduction in the number of stressors by optimizing management and housing conditions might be a way to reduce tail biting, one huge challenge in modern pig production [21]. A study of Gentz et al. [22] examined docked and undocked pigs that were either conventionally housed after weaning or remained in so-called wean-to-finish housing (W-F). The reduced regrouping event at the beginning of fattening period benefited the W-F pigs in terms of tail lesions and losses, but still tail lesions and losses occurred and the authors concluded that further optimization of housing systems are necessary. Although routine tail docking is prohibited in the EU since 1994 (Council Directive 2008/120/EC), an audit that was carried out in Germany in 2018 reported that 95% of conventionally housed pigs were tail-docked [23] and it is still one of the most commonly used measures to reduce tail biting in pigs [21,24]. As action plans of the concerned EU member states were initiated to assist farmers in the housing of undocked pigs, substantiating research of tail biting behavior of pigs is needed. To the authors’ knowledge, almost no scientific publications are available concerning rearing in the farrowing pen. The previous studies of Gentz et al. [22] and Lange et al. [10] showed the potential of alternative housing conditions, but both stated that further investigations are needed as, for example, Lange et al. [10] did not study long-term effects. Long-term effects of the housing system during suckling period on the regrouping behavior at the beginning of the fattening period are scarcely studied.

Therefore, the aim of this study was to investigate the effects of different housing systems during suckling period followed by rearing in the same system on skin and tail lesions, tail losses and performance of docked and undocked growing and finishing pigs. We hypothesized that piglets from loose-housed sows, either from single loose-housing or group housing, react milder on the weaning stressors by showing less skin lesions, less tail lesions and, therefore, less tail losses. Rearing in the farrowing pen reduces relocation and regrouping at weaning; therefore, we hypothesized a decreased incidence of skin lesions, tail lesions and, consequently, tail losses in this rearing system, especially in regard to the housing of undocked pigs. As cross-suckling can occur in group-housing systems of lactating sows, we further hypothesized that the average daily gain of piglets from group housing can be impaired, compared to gains of piglets from single housing systems such as farrowing crates or single loose-housing. We also hypothesized that early socialization during the lactation period lowers the incidence of skin lesions during a late first regrouping for fattening period.

## 2. Materials and Methods

### 2.1. Animals and Housing

**Figure 1:**
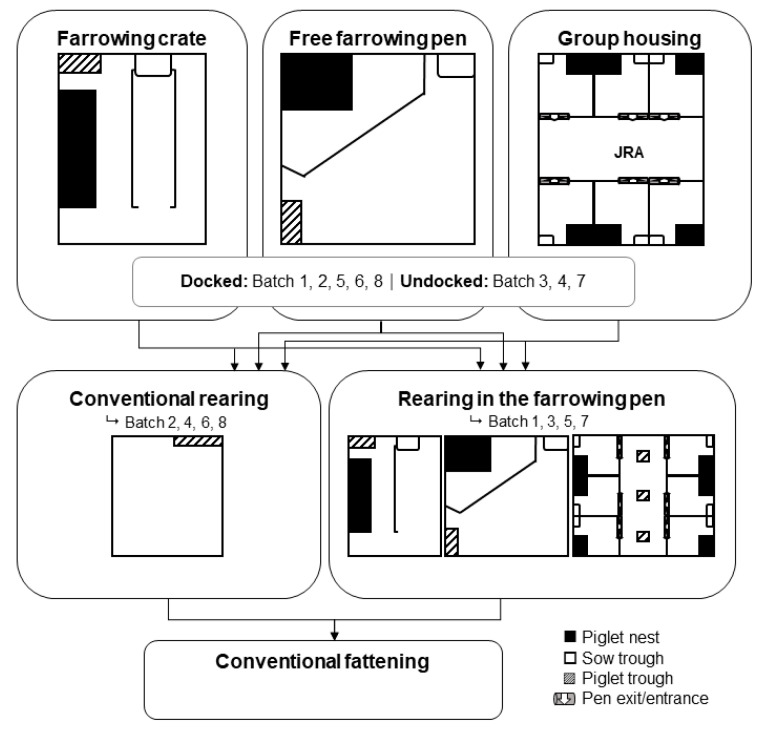
Schematic overview of the experimental design (JRA: joint running area; drawings are not to scale).

The study was conducted on the experimental farm for pigs of the Chamber of Agriculture for Lower Saxony in Wehnen, Germany. In eight batches between December 2016 and January 2018, in total 1106 crossbred (Piétrain × [Landrace × Large White]) pigs were included in a 3 × 2 × 2 factorial design study from three different farrowing systems: (1) conventional single-housing in farrowing crates (FC; *n* = 349), (2) single-housing in free farrowing pens (FF; *n* = 340), and (3) group housing of lactating sows (GH; *n* = 417) (Figure 1).

All farrowing systems were located in the same building and for each one two compartments were used. FC compartments included eight pens, GH and FF compartments included six pens each. The FC pens measured 2.6 × 2.0 m^2^ and had tiles in the lying area of the sow, the rest was a fully slatted plastic floor (slat width = 10 mm, tread area = 11 mm). The open creep area was removed for rearing. The FF pens measured 2.7 × 2.7 m^2^. The creep area in the FF pens was a box (1.0 × 0.8 m^2^) with two openings which were closed with the proceeding rearing period. The flooring of the FF pen was similar to that in the FC pens. In each of the two compartments of GH, six sows were stalled in single-housing free-farrowing pens until day 6–7 postpartum. Then, single-pens were opened and sows and their litters were able to interact freely in a joint running area (JRA) until weaning. The pens measured 2.0 × 2.5 m^2^ each, the joint running area measured 6.1 × 2.4 m^2^. The flooring in the GH pens was a partly fully slatted concrete floor (slat width = 10 mm, tread area = 90 mm) and partly fully slatted cast-iron floor (slat width = 11 mm, tread area = 15 mm). The creep areas were similar to those in the FF pens. The floor in the JRA was a fully slatted concrete floor (slat width = 10 mm, tread area = 90 mm). Nipple drinkers for sows and piglets were located in the pens and in the JRA. Sows were moved simultaneously to the farrowing system seven days before the expected farrowing date. Sows in FC were confined permanently during farrowing and suckling period. Sows in FF and GH could move freely and interact with their piglets for the whole suckling period of 26.6 ± 1.5 days (for further details on farrowing pens, see [17]). In all three farrowing systems, weaning took place on the same day. Until day 4 after birth, all male piglets were castrated and, batchwise, tails were either docked under veterinary advisement using a hot cautery iron or left intact. Piglets were weaned batchwise to one of two different rearing systems: either (1) piglets were relocated and regrouped into conventional rearing pens (Conv; *n* = 486) or (2) piglets remained in their farrowing system (Reaf; *n* = 620) for the rearing period. In conventional rearing, a maximum of 23 piglets per equally balanced mixed-sex group were housed on fully slatted plastic floors with a minimum space allowance of 0.37 m^2^ per pig (Table 1). In Reaf housing, piglets remained together with their littermates for rearing. Since piglets were not relocated or regrouped for the rearing period, cross-fostering was necessary to guarantee the legally required space-allowance during rearing. Within the first 48 h after birth, FC and FF litters had to be adjusted to a maximum of 14 piglets per pen. Therefore, and due to suckling piglet mortality (FC = 12.3%; FF = 25.6%; GH = 19.9%; see [25]), the space allowance differed between the Reaf systems from 0.37 to 1.46 m^2^ per pig (Table 1).

In both rearing systems, the same commercial diet OlymPig^®^ (Agravis Raiffeisen AG, Münster, Germany) was fed ad libitum and manipulable material such as pieces of wood and ropes were provided (one piece of wood and one rope per pen) and renewed continuously. After 37.5 ± 0.5 days of rearing, piglets were relocated, regrouped and sorted by sex for the fattening period. Due to a limited number of identically constructed fattening pens, not all rearing pigs could be studied during fattening—a subset of 791 pigs was moved to the fattening compartments. Batchwise, fattening took place in one of three compartments (compartment I: 18 pigs per pen with a space allowance of 0.88 m^2^ per pig; compartment II: 18 pigs per pen with a space allowance of 0.99 m^2^ per pig; compartment III: 8 pigs per pen with a space allowance of 0.96 m^2^ per pig). Fattening pigs were housed on fully slatted concrete floors, fed ad libitum and manipulable material such as pieces of wood and ropes (each one per pen) were provided. With an average live weight of 122.7 ± 3.9 kg, which was reached within 159.7 ± 11.9 days of life, pigs were taken to slaughter. Tail-docking was the third factor in the study design. When severe tail lesions (see Section 2.2.2) occurred, manipulable material was renewed, and jute bags and hayracks were additionally installed into the pens. A climate computer regulated ventilation and heating in both rearing systems, starting with an air temperature of 28 °C on the first days after weaning. In addition to diffuse daylight, artificial lighting was provided for 10 h between 7:30 a.m. and 5:30 p.m. at all farm stages.

### 2.2. Recorded Traits

#### 2.2.1. Scoring of Skin Lesions

**Figure 2:**
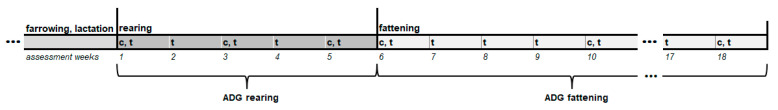
Timeline for the assessed and analyzed parameters over the 18 assessment weeks (c: complete assessment of skin lesions, ear lesions and losses, lameness and manure on the body; t: assessment of tail lesions and losses; ADG: calculated average daily gain).

All growing (*n* = 1106) and finishing pigs (*n* = 791) were assessed for skin lesions starting on the first day after weaning, following the Welfare Quality^®^ assessment protocol applied to growing and finishing pigs [26]. The assessment was carried out by two assessors who were trained together. For each assessment, the two persons were present; one being the assessor, the other one an active observer, who justified the given score. The same scoring approach for the three-staged lesion score was used, as published by Lange et al. [10]. During the rearing period, pigs were scored every two weeks for skin lesions. During the fattening period skin lesions were assessed every four weeks (Figure 2).

In total, skin lesions were primarily found on the ears (score 1 = 14.7%; score 2 = 4.6%), front (score 1 = 37.1%; score 2 = 10.5%), and middle part (score 1 = 22.2%; score 2 = 4.1%) and to a negligible amount on hind-quarters (score 1 = 8.9%; score 2 = 0.8%) and legs (score 1 = 1.3%; score 2 = 0.2%) (frequencies calculated with the FREQ procedure in SAS^®^ (SAS Institute Inc., Cary, NC, USA)). Due to the very low occurrence of severe lesions, scores were summarized into a binary trait, (0 = no lesions and 1 = skin lesions) for the statistical analysis. As skin lesions after rank fights commonly appear on the anterior body parts, lesions on the first three body parts were statistically analyzed [7,27]. Assessment weeks 1 (first assessment after weaning), 5 (end of rearing), 6 (first assessment after regrouping for fattening) and 18 (end of fattening) were included in the analysis to evaluate the crucial periods for skin lesions [4].

#### 2.2.2. Scoring of Tail Lesions and Tail Losses

Starting one day after weaning, the tails of all pigs were scored individually weekly for 18 weeks using a modified key, described by Abriel and Jais [28] (Figure 2). Score 0 was given for intact tails without scratches or bite marks. Slight scratches or minor bite marks were scored as “a lesion” (score 1). A “severe lesion” was defined by deeper, flat lesions (score 2) and a “very severe lesion” was greater than 2 cm with deep, flat lesion (score 3) [22]. For the statistical analysis, the tail lesion score was summarized due to the low occurrence of severe and very severe lesions (<2.6%) into “no lesions” (score 0) and “lesions” (score 1–3). Tail losses were scored in quarters: “original length” (no loss; score 0), “tip loss” (max. ¼ lost; score 1), “half loss” (max. ½ lost; score 2), “major loss” (max. ¾ lost; score 3), and “complete loss” (more than ¾ lost, score 4) [29]. For the statistical analysis, these scores were also summarized into “intact tail” (score 0) and “tail loss” (score 1–4). Due to a low occurrence of tail losses in docked pigs, only data of undocked pigs went into analysis. To outline the progression of the tail losses, tail losses of assessment weeks 1 (beginning of rearing period), 5 (end of rearing), 10 (middle of fattening) and 18 (end of fattening) were calculated.

#### 2.2.3. Performance

The individual body weights of all pigs were measured at weaning, at the end of the rearing period and 2.8 ± 1.2 days prior to marketing/slaughter (scales from T.E.L.L.-Steuerungssysteme GmbH & Co. KG, Vreden, Germany) and the average daily gain (ADG) was calculated separately for the rearing and fattening period in gramm per day (g/d) (Figure 2).

#### 2.2.4. Other Measures

Additionally, pigs were scored for ear lesions, ear losses, lameness and manure on the body every two weeks during the rearing period. During the fattening period these parameters were assessed every four weeks (Figure 2). Ear lesions and losses were scored with the ‘German Pig Scoring Key’ (German: ‘Deutscher Schweine-Boniturschlüssel’; [30]). Lameness and manure on the body were scored following the Welfare Quality^®^ assessment protocol applied to growing and finishing pigs [26]. Since the majority of animals was scored 0 for all of these parameters, no statistical analysis was performed (score 0: ear lesions = 99.1%; ear losses = 99.5%; lameness = 98.1%; manure on the body = 91.0%; frequencies calculated with the FREQ procedure in SAS^®^ (SAS Institute Inc., Cary, NC, USA)).

### 2.3. Statistical Analysis

The data analysis was conducted with SAS^®^ 9.4 (SAS Institute Inc., Cary, NC, USA). Due to the non-normally distributed data of skin lesions, tail lesions and tail losses, a general linear model assuming a binomial distribution was applied using the GLIMMIX procedure. For each parameter, all possible fixed effects were tested and only included in further analysis if parameters showed significant influence on the model (*p* < 0.05) and decreased the ‘Akaike’s information criterion corrected’ (AICC; [31]) and ‘Bayesian information criterion’ (BIC; [32]). The model with the lowest values for AICC and BIC was used for further evaluation. Due to the study design and management routines, it was not possible to house Conv and Reaf, and docked and undocked pigs simultaneously per batch during rearing and fattening period. Therefore, the batch effect (batch 1–8) was considered as a random effect.

Each model for the skin lesion scores included the fixed effects farrowing system (FC, FF, GH), rearing system (Conv, Reaf) and assessment week (1, 5, 6 and 18). Sex (male, female) was only significant for the model of the skin lesions on the front body part and thus included into the respective model. For the front and ears model, the interaction of rearing system × farrowing system × assessment week was tested as significant and, therefore, included; for the middle body part, the interaction of farrowing system × assessment week was tested as significant. Batch and animal identities were added as random effects.

The model for the prevalence of tail lesions included the fixed effects rearing system (Conv, Reaf), docking status (docked, undocked), assessment week (1–18) and the interaction of rearing system × docking status × assessment week. Batch and animal identities were added as random effects.

The fixed effects farrowing system (FC, FF, GH), rearing system (Conv, Reaf), assessment week (1, 5, 10 and 18), sex (male, female) and the interaction of rearing system × assessment week were included in the model of tail losses. Batch and animal identities were added as random effects.

The data on average daily gain (ADG) were analyzed in two models, one for rearing and one for fattening period, using the MIXED procedure in SAS^®^ 9.4 (SAS Institute Inc., Cary, NC, USA). Both models included the fixed effects farrowing system (FC, FF, GH), docking status (docked, undocked) and their interaction (docking status × farrowing system). The model for the fattening period included sex (male, female) as significant fixed effect. The weight at the beginning of each period was used as a covariable to take the different initial weights of the respective animals into account. Batch and animal identities were added as random effects. Residuals fulfilled requirements to assume variance homogeneity as well as a normal distribution.

For all models, the significance of differences in the least square means was adapted by the Bonferroni-correction to adjust for multiple comparisons.

## 3. Results

### 3.1. Skin Lesions

For all three body parts, the farrowing system and the assessment week had a significant effect on skin lesions of growing and finishing pigs (*p* < 0.05). The rearing system affected skin lesions on the front and ears significantly (*p* < 0.05) but not on the middle (*p* > 0.05). For the front and ears, the interaction of rearing system × farrowing system × assessment week was significant (*p* < 0.05) and the interaction of farrowing system × assessment week affected the skin lesion score on the middle body part. Sex only affected skin lesions on the front body (*p* < 0.05).

**Figure 3:**
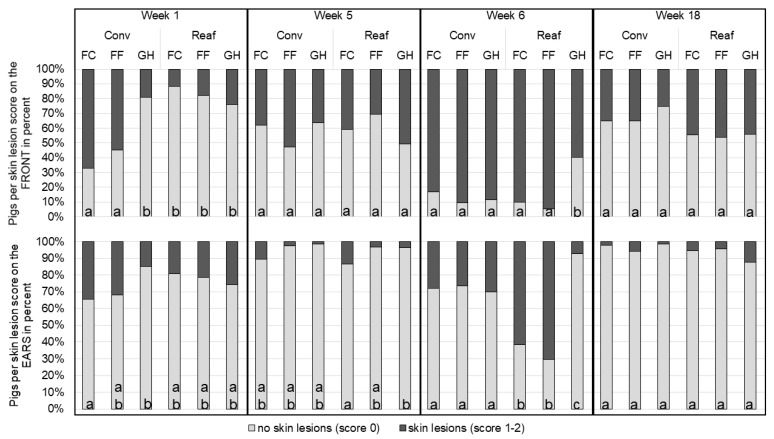
Estimated frequencies of skin lesions in percentages for the interaction rearing system × farrowing system × assessment week per body part (Conv: conventional rearing; Reaf: rearing in the farrowing pen; FC: farrowing crate; FF: free farrowing pen; GH: group housing of lactating sows; a–c: different letters indicate significant differences of score 0 (no lesions) within week and body part (*p* < 0.05)).

In week 1, significantly fewer Conv-GH pigs had skin lesions on the front body than Conv-FC and Conv-FF pigs (*p* < 0.0001) and on the ears than Conv-FC pigs (*p* = 0.0316; Figure 3). Pigs from Conv-GH did not differ from pigs raised in the farrowing pen (*p* = 1.0). Pigs from Reaf did not significantly differ among the farrowing systems (*p* > 0.6). Numerically, more Reaf-GH pigs had skin lesions on the front and ears than Reaf-FC or Reaf-FF pigs. On the middle body part, significantly more pigs born in FC had skin lesions than pigs born in GH in week 1 (FC = 9.0%^a^; FF = 6.6%^ab^; GH = 2.8%^b^; *p* = 0.0272).

In week 5, at the end of the rearing period, no significant differences between the groups could be detected on the front and the middle (*p* > 0.05) but, on the ears, less Reaf-GH pigs had lesions than Reaf-FC pigs (*p* = 0.048). In week 6, all groups showed the highest occurrence of skin lesions on the front body. Conv Pigs did not significantly differ in the number of skin lesions (*p* = 1.0). On the front and the ears, significantly less Reaf-GH pigs had skin lesions than the other experimental groups (*p* < 0.03). Reaf-FC and Reaf-FF did not differ from the Conv pigs concerning skin lesions on the front body (*p* = 1.0). On the middle body part, significantly less GH and FC pigs had skin lesions than FF pigs in week 6 (FC = 65.9%^a^; FF = 82.1%^b^; GH = 50.1%^a^; *p* < 0.02). In week 18, at the end of fattening period, no significant effect of the housing systems could be found (*p* > 0.69) (pigs with skin lesions on middle body part: FC = 12.3%; FF = 7.7%; GH = 11.5%).

Within the housing systems Conv-FC, Conv-FF, Conv-GH, Reaf-FC and Reaf-FF a significant increase in the number and severity of skin lesions could be found between the end of rearing (assessment week 5) and the beginning of fattening (assessment week 6) (*p* < 0.0001), except for the housing system Reaf-GH. Only Reaf-GH pigs showed no significant difference in skin lesions on the front body part and the ears after regrouping for fattening (*p* = 1.0). Significantly more female than male pigs had skin lesions on the front body (Score 1: 53.7% vs. 48.5%; *p* = 0.0272).

### 3.2. Tail Lesions and Tail Losses

The rearing system, docking status, assessment week and the interaction of rearing system × docking status × assessment week significantly affected the incidence of tail lesions (*p* < 0.05). Significantly less docked pigs had tail lesions than undocked pigs (docked = 7.4%; undocked = 15.8%; *p* < 0.0001).

**Figure 4:**
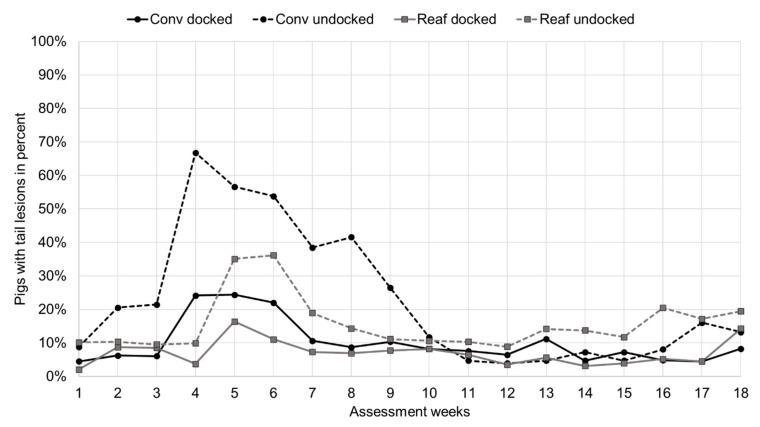
Frequency in the percentage of back transformed tail lesion estimates for docked and undocked pigs per rearing system over 18 assessment weeks (Conv: conventional rearing, Reaf: rearing in the farrowing pen). For a better clarity of the figure, significances are shown in Table 2.

In week 1 of the rearing period, Conv and Reaf, docked and undocked, pigs started with around the same level of tail lesions (Figure 4; Table 2). In a range from 2.1% (docked Reaf) up to 10.2% (undocked Reaf) of the animals suffered from tail lesions in the beginning. In week 2, the number of undocked Conv pigs with tail lesions increased (20.5%), while undocked Reaf pigs kept the preceding level of tail lesions (10.4%). Conv undocked pigs reached a peak in tail lesions in week 4 of the rearing period (66.8%). Two weeks later, in assessment week 6, Reaf undocked pigs reached their maximum in tail lesions (36.2%). Tail lesions of docked Conv pigs peaked in week 4 (24.2%) and of docked Reaf pigs in week 5 (16.4%). After reaching the peak, the estimated frequency of tail lesions declined over the fattening period, for undocked Conv as well as Reaf pigs. Through most of the assessment weeks, undocked Conv and undocked Reaf pigs did not differ significantly (*p* > 0.05), except for week 4 and 8 where significantly more Conv pigs had tail lesions than Reaf pigs (*p* < 0.02). Still, docked and undocked pigs within their housing systems did differ significantly (*p* < 0.05; Table 2). While less docked Conv pigs had consecutively, from week 2 until week 8, less tail lesions than undocked Conv pigs, equivalent differences for Reaf pigs could only be found in weeks 5, 6 and 16.

The amount of tail losses of undocked pigs was affected significantly by the rearing system, the farrowing system, assessment week, sex and the interaction of rearing system × assessment week (*p* < 0.05).

**Figure 5:**
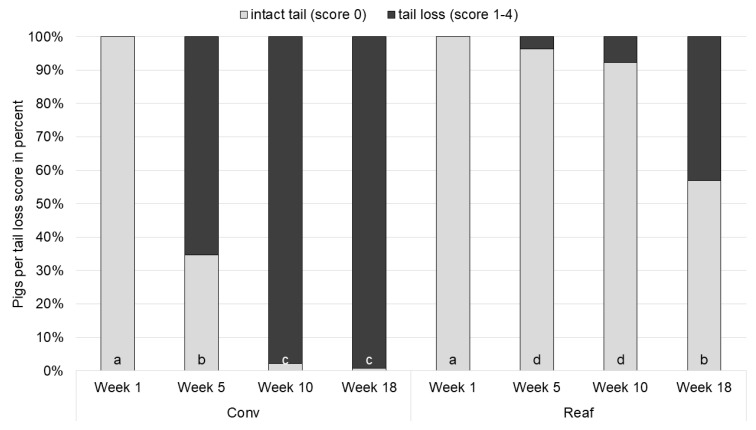
Frequency in percent of back transformed tail loss estimates of the interaction of rearing system × assessment week of undocked pigs (Conv: conventional rearing; Reaf: rearing in the farrowing pen; a–d: different letters indicate significant differences of score 0 (no losses) across housing system and assessment week (*p* < 0.05).

In the first assessment week, no significant differences between the housing systems could be found (Figure 5). In both rearing systems, 100.0% of the pigs started with intact tails. At the end of the rearing period (week 5), two thirds of Conv animals (65.3%) lost parts of their tails, while 96.3% of Reaf pigs still had intact tails (*p* < 0.0001). In the middle, and the end of fattening (week 10 and week 18), few Conv pigs still had an intact tail. In comparison, 43.1% of Reaf pigs lost part of their tails before the end of fattening. Concerning the impact of the farrowing system, significantly more FC pigs had intact tails than GH pigs (intact tails: FC = 94.4%^a^; FF = 88.7%^ab^; GH = 65.7%^b^; *p* = 0.0021). In any case, 77.9% of males and 91.9% of female pigs had intact tails (*p* = 0.0219).

### 3.3. Performance

In the rearing and fattening period, the average daily gain was significantly affected by the docking status, the farrowing system and their interaction docking status × farrowing system (*p* < 0.05). The ADG in fattening period was affected significantly by sex (*p* < 0.05).

**Figure 6:**
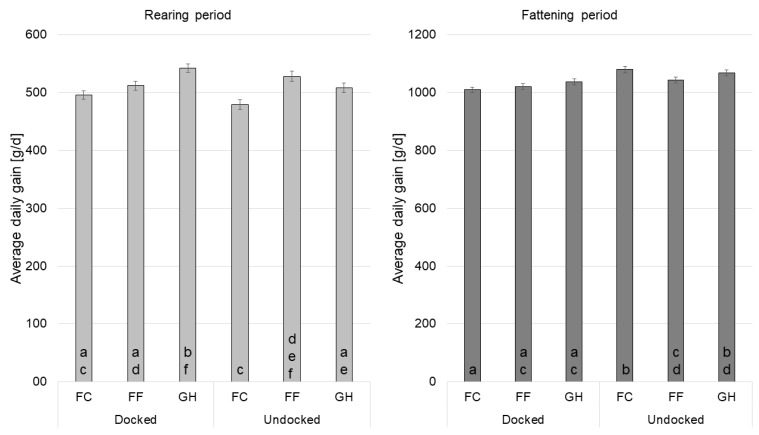
Estimated average daily gain in gram per day for the interaction of docking status × farrowing system (FC: farrowing crate; FF: free farrowing pen; GH: group housing of lactating sows; a–f: different letters indicate significant differences within the rearing or fattening period (*p* < 0.05)).

Docked pigs gained 11 g/d more than undocked pigs (516.4 g/d vs. 505.0 g/d, respectively; *p* = 0.0147) during rearing period. In fattening period, undocked pigs had higher ADG than docked pigs (1063.2 g/d vs. 1022.0 g/d; *p* < 0.0001). With 487.1 g/d, pigs born into FC had significantly lower ADG during rearing than pigs from FF and GH (FF = 519.9 g/d, GH = 524.9 g/d; *p* < 0.0001). During fattening period, pigs born in GH had significantly higher ADG than FF pigs (FC = 1044.2 g/d^ab^; FF = 1031.6 g/d^a^; GH = 1052.1 g/d^b^; *p* = 0.0227). Considering the interaction of docking status and farrowing system, docked GH pigs gained significantly more weight daily than docked FC and FF pigs (*p* < 0.001) during rearing period (Figure 6). In the same period, undocked FC pigs had significantly lower ADG than undocked FF and GH pigs (*p* < 0.02). In fattening period no significant differences could be detected between docked pigs (docked FC = 1008.9 g/d; docked FF = 1020.5 g/d; docked GH = 1036.6 g/d; *p* > 0.09). Undocked FC fattening pigs gained significantly more than undocked FF fattening pigs (*p* = 0.0208). Males gained significantly more weight daily than females during fattening period (1067.7 g/d vs. 1017.5 g/d; *p* < 0.0001).

## 4. Discussion

Up to now only little scientific knowledge is available concerning the rearing in the farrowing pen and the long-term effect of the farrowing system on the regrouping behavior at fattening period. Especially the effects of rather late first regrouping for fattening on skin lesions, tail lesions and losses and performance was up to now not studied.

### 4.1. Skin Lesions

In accordance with a previous study of Lange et al. [10] and our first hypothesis, mainly the farrowing system affected the occurrence of skin lesions at regrouping occasions (here: week 1 and week 6) with GH piglets showing fewer skin lesions after weaning and regrouping for fattening than piglets born in FF or FC. According to the findings of Lange et al. [10] but against our hypothesis, FF piglets in the present study did not fully benefit from the loose farrowing system, showing no differences in skin lesions compared to FC pigs. In week 1, significantly more Conv-GH pigs were scored 0 for skin lesions than Conv-FF and Conv-FC pigs. In comparison, Reaf-GH pigs had less skin lesions at their first regrouping event in week 6, than their counterparts born and reared in the FF and FC pens. Likewise, Bohnenkamp et al. [9] found that GH pigs had fewer scratches after weaning than piglets of single housed sows. They also found a decreased number of fights and fighting duration in GH pigs. Hessel et al. [8], who observed the behavior of piglets that were co-mingled during the lactation period, noticed that early socialized piglets showed less fighting behavior than control groups, especially in the first 4 h after weaning. D’Eath [7] showed that mixing litters prior to weaning even had long-term effects on the social behavior of the pigs: when the experimental pigs were mixed again after weaning, they formed the new stable hierarchy more rapidly. All these findings were confirmed in a study of Kutzer et al. [11] who also found that co-mingled piglets had less skin lesions than single-housing piglets that had no contact to other litters. They further conclude that the differences in skin lesions must originate from a difference in severity in fights for hierarchy. Direct behavioral observations were not done in the present study, but, as Turner et al. [33] and Bohnenkamp et al. [9] assumed, the assessment of skin lesions can be used to determine the intensity of agonistic behavior of pigs. To conclude, it can be assumed that GH pigs in the present study fought less and/or for a shorter period after regrouping at weaning and after rearing in the farrowing pen (Reaf-GH) and had consequently fewer skin lesions than (Conv and Reaf) FF and FC pigs. In weeks 5 and 18, when animals were familiar with each other and their housing conditions, no significant differences between the experimental groups could be detected. Still, the average number of skin lesions on the front body part was rather high. This may be due to the decreasing space allowance, as these time points mark the end of each period (growing, fattening). This is in accordance with findings of Ewbank and Bryant [3], who showed that the decrease in space increased the occurrence of agonistic behavior within the pig group. In summary, the present work confirms that the co-mingling of piglets during the suckling period has immediate advantages on the incidence of skin lesions after regrouping at weaning. Nevertheless, early socialization before weaning had no long-term effect on a second regrouping at fattening (Conv).

### 4.2. Tail Lesions and Tail Losses

As expected, the docking status had a strong impact on the incidence of tail lesions and tail losses. Although routine tail docking is prohibited in the EU since 1994 (Council Directive 2008/120/EC), it is still one of the most commonly used measures to reduce tail biting in pigs [21,23,24]. However, this is ethically questionable and under discussion in many countries. In the present study, tail losses were primarily observed on the tails of undocked pigs and only to a negligible amount on tails of docked pigs, which is why the latter were excluded from the statistical analysis, as done in other studies, e.g., Abriel and Jais [28] and Gentz et al. [22].

Despite the docking status, the interaction of rearing system, docking status and assessment week affected the incidence of tail lesions significantly. According to our hypothesis, undocked animals reared in the farrowing pen had significantly fewer tail lesions than undocked pigs that were regrouped and rehoused for rearing. The weaning process, often accompanied by mixing, is considered as the first major stressful event in a piglet’s life [1]. Regrouping inevitably leads to hierarchical fights, causing skin injuries and negatively affecting animal welfare [3,5,7,9,10]. In the present study, undocked piglets reared in the farrowing pen in particular seemed to benefit from the absence of regrouping at weaning. Likewise, a study of Gentz et al. [22] showed that tail lesions were reduced and remained on a lower level for undocked pigs which were housed under reduced regrouping conditions. Besides the reduced regrouping at weaning, rearing in the farrowing pen and conventional rearing differed in space allowance and group size during the rearing period. As already mentioned, the decrease in space allowance increased the amount of aggressive behavior of pigs in a study conducted by Ewbank and Bryant [3]. Gentz et al. [22] also found fewer tail lesions and losses in the housing system with a greater space allowance. These findings can be confirmed by the present study, leading to the assumption that the reduction in the number of external stressors, such as regrouping and space allowance, can reduce the incidence of tail lesions and consequently of tail losses. It remains to be mentioned that the difference in space allowance in Reaf housing results from higher piglet losses, especially in the FF and GH housing systems [25]. Higher piglet loss should not only be considered in welfare matters, but also taken into account in economic considerations. Additionally, the feasibility of rearing in the farrowing pen should be discussed. In short, to practice rearing in the farrowing pen, farmers will need to rethink their housing systems. Rearing pens would become redundant, instead more farrowing pens should be installed to maintain the previous workflow. This might create costs, which then again might be saved through less management measures as, for example, one cleaning step is pared down. More studies, especially from an economic point of view, are needed.

As the third major factor which affected the incidence of tail lesions, the assessment week should be discussed. In the present study, the frequency of tail lesions slightly rose for conventionally reared undocked pigs around week 2, culminating in week 4. For undocked pigs reared in the farrowing pen tail lesions increased in week 5 and culminated in week 6. Generally, it is common for undocked pigs to start tail biting or the so-called tail-in-mouth behavior in the first two weeks after weaning [22,28,29,34]. In accordance to the present findings, these studies showed that undocked pigs in enriched environments started to bite later in time compared to conventionally housed undocked pigs. Naya et al. [29] and other studies also discussed the provision of enrichment material and showed that the offering of organic manipulable material such as straw [24], peat [35] or straw–peat mixtures [29,36] influences the behavior of the piglets positively in terms of less aggressive biting [35] and less tail losses [36]. Still, all authors state that the sole provision of organic enrichment material over a limited time period cannot eliminate welfare altering behavior completely.

The farrowing system did not affect the frequency of tail lesions, so it seems that acute housing conditions have a greater impact on tail lesions than former housing conditions during lactation or early socialization [22,28]. Still, the farrowing system affected the extent of individual tail loss. Significantly more pigs born in GH suffered from tail loss than pigs born in farrowing crates or free farrowing pens. Gentz et al. [22] who examined similar farrowing systems, found no significant but numerical differences between the farrowing systems concerning tail loss. In their study, GH pigs also had more tail losses after rearing period than pigs from the other two farrowing systems. The fact that our results show that GH pigs did not have more tail lesions, but still more tail losses than the other groups might be explained by the type of biting behavior. On one hand, direct behavioral observations could have brought more details on the individual biting behavior [37]. GH pigs might have bitten each other in a more damaging way than FF and FC pigs. On the other hand, another explanation could be the condensation of the scoring keys for tail lesions and tail losses. Tails of GH pigs were slightly more often scored with severe and very severe tail lesions than tails of FF and FC pigs. In fact, tails of GH pigs were also scored more often with higher tail loss scores than FF and FC pigs, but due to the overall low occurrence of severe tail lesions and large tail losses this could not be presented in the results.

### 4.3. Performance

The average daily gain in this study was affected by the docking status and the farrowing system. While docked pigs had higher daily gains than undocked pigs during rearing, undocked pigs had higher gains during fattening period. A study of Sinisalo et al. [38] found significant differences in the ADG between victims and non-victims of tail biting. Victims of tail biting gained 33.4 g/d less than non-victims. Pigs in this study were not classified as victims or non-victims, and no data are presented on whether all undocked pigs were bitten at least once. Since tail lesions in the present study occurred with the highest frequency at the end of rearing, it is likely to have many victims of tail biting in this period. This may explain that undocked pigs had lower ADG during the rearing period than docked pigs. After regrouping for fattening, all animals were housed under similar conditions and undocked pigs obviously managed to overcome the preceding impaired ADG resulting in higher ADG of undocked pigs during fattening period. Sinisalo et al. [38] stated that there were other more distinct effects on ADG than tail biting, i.e., sex and breed. Since pigs in the present study belonged to one breed, no breed effect could be examined, but the ADG in the present study differed significantly between females and barrows. This well agrees with findings of Collins et al. [18] who found higher ADGs in male than in female pigs during the finisher period.

Against our hypothesized expectations of impaired weight gain due to cross-suckling, pigs born in GH had comparably high ADGs during rearing and fattening, irrespective whether docked or undocked. Morgan et al. [16] found a rather low rate of cross-suckling in their study (average 2.9% alien piglets on total suckling events) and no housing effect on the weight at weaning of the piglets. In the study of Nicolaisen et al. [17], which was conducted in the same project and, therefore, done with partly the same animals as the present study, at least one alien piglet could be detected at a sow’s udder in 35% of all suckling bouts. Still, GH pigs had significantly higher ADG than FC piglets in the present study, especially during rearing. Hessel et al. [8] who studied co-mingling during lactation also found a tendency of GH pigs to gain more weight. The authors stated that early socialized piglets seem to be better prepared to overcome the usual growth lag after weaning. In the study of Kutzer et al. [11], piglets born in farrowing crates had significantly lower body weight gain over the first four days after weaning compared to loose-housing and group-housing piglets. In accordance to the present study, weight gain was highest in GH piglets and the lowest in FC piglets. In contrast, D’Eath [7] could not detect differences in growth between treatments following mixing.

### 4.4. Other Measures

Measures for ear lesions, ear losses, lameness and manure on the body were assessed, but not statistically analyzed due to an overall very low occurrence. Lühken et al. [39] examined air hygiene during suckling period in the same three farrowing systems on the same research farm as described in the present study. In their study, they conclude that FF and GH not only offered better welfare for the sows through free movement, but also acceptable air quality. These findings of good hygiene can be augmented by our findings that the majority of pigs in this study did not show manure on the body, although raised consecutively in the farrowing system.

## 5. Conclusions

Piglets housed in a group housing system during lactation profited from co-mingling and early socialization, resulting in lower incidences of skin lesions after regrouping events, especially for a late first regrouping for the fattening period. Piglets born in free farrowing pens showed only marginal benefits on the examined parameters compared to piglets raised in pens with a farrowing crate. The incidence of tail lesions and losses was mainly affected by the acute housing during rearing and fattening than by the housing system during lactation. The reduced regrouping as part of rearing in the farrowing pen resulted in positive effects on the incidence of tail lesions and losses in undocked pigs. Group housing and rearing in the farrowing pen did not negatively affect the performance during rearing or fattening. Nevertheless, the high piglet mortality during suckling period in the two free farrowing systems (FF and GH) should be reduced before a clear recommendation can be given.

## Figures and Tables

**Table 1:** Mean, minimum, maximum and standard deviation (SD) of space allowance per pig in square meters [m^2^] and of group size per rearing and farrowing system during rearing period.

Rearing System	Farrowing System	N Pens	Space Allowance				Group Size			
			Mean	Min	Max	SD	Mean	Min	Max	SD
Conv	FC	8	0.44	0.37	0.77	0.14	20.50	11.00	23.00	4.00
	FF	8	0.49	0.37	1.21	0.29	19.88	7.00	23.00	5.36
	GH	8	0.42	0.37	0.53	0.06	20.25	16.00	23.00	2.49
Reaf	FC	16	0.46	0.37	0.65	0.08	11.50	8.00	14.00	1.79
	FF	19	0.83	0.56	1.46	0.28	9.53	5.00	13.00	2.50
	GH	24 *	0.71	0.67	0.77	0.05	63.50	58.00	67.00	4.04

*: 24 pens reared in 4 groups; SD: standard deviation; Conv: conventional rearing; Reaf: rearing in the farrowing pen.

**Table 2:** Multiple comparison of the least-square means differences of the interaction assessment week × rearing system × docking status by Bonferroni within the assessment week (*p* < 0.05).

	Assessment Week																	
	1	2	3	4	5	6	7	8	9	10	11	12	13	14	15	16	17	18
Conv docked	a	a	a	a	ac	ac	a	a	a	a	a	a	a	a	a	a	a	a
Conv undocked	a	b	b	b	b	b	b	b	a	a	a	a	a	a	a	ab	ab	a
Reaf docked	a	ab	ab	c	a	a	a	a	a	a	a	a	a	a	a	a	ab	a
Reaf undocked	a	ab	ab	c	bc	bc	ab	a	a	a	a	a	a	a	a	b	b	a

Conv: conventional rearing; Reaf: rearing in the farrowing pen; a–c: Significant differences within the assessment week (*p* < 0.05).

## Data Availability

None of the data were deposited in an official repository. The generated and analyzed data sets used in the current study are available from the corresponding author on reasonable request.
